# The Enigmatic N-Terminal Domain of Proglucagon; A Historical Perspective

**DOI:** 10.3389/fendo.2021.683089

**Published:** 2021-06-11

**Authors:** J. Michael Conlon

**Affiliations:** Diabetes Research Group, School of Biomedical Sciences, Ulster University, Coleraine, United Kingdom

**Keywords:** glucagon, enteroglucagon, glicentin, glicentin-related pancreatic peptide, radioimmunoassay

## Abstract

Enteroglucagon refers to the predominant peptide with glucagon-like immunoreactivity (GLI) that is released by the intestine into the circulation in response to nutrients. Development of a radioimmunoassay for glucagon revealed issues that were not apparent in applications of the insulin radioimmunoassay. The fact that some antisera raised against glucagon recognized glucagon-related peptides in extracts of both pancreas and gut whereas others recognized only components in the pancreas remained a mystery until it was realized that the “gut GLI cross-reactive” antisera were directed against an epitope in the N-terminal to central region of glucagon whereas the “pancreatic glucagon specific” antisera were directed against an epitope in the C-terminal region. Unlike the cross-reactive antisera, the glucagon specific antisera did not recognize components in which glucagon was extended from its C-terminus by additional amino acids. Initial attempts to purify enteroglucagon from porcine ileum led to the erroneous conclusion that enteroglucagon comprised 100 amino acids with an apparent molecular mass of 12,000 Da and was consequently given the name glicentin. Subsequent work established that the peptide constituted residues (1-69) of proglucagon (M_r_ 8128_)_. In the 40 years since the structural characterization of glicentin, attempts to establish an unambiguous physiological function for enteroglucagon have not been successful. Unlike the oxyntomodulin domain at the C-terminus of enteroglucagon, the primary structure of the N-terminal domain (glicentin-related pancreatic peptide) has been poorly conserved among mammals. Consequently, most investigations of the bioactivity of porcine glicentin may have been carried out in inappropriate animal models. Enteroglucagon may simply represent an inactive peptide that ensures that the intestine does not release equimolar amounts of a hyperglycemic agent (glucagon) and a hypoglycemic agent (GLP-1) after ingestion of nutrients.

## Introduction

**Figure 1:**
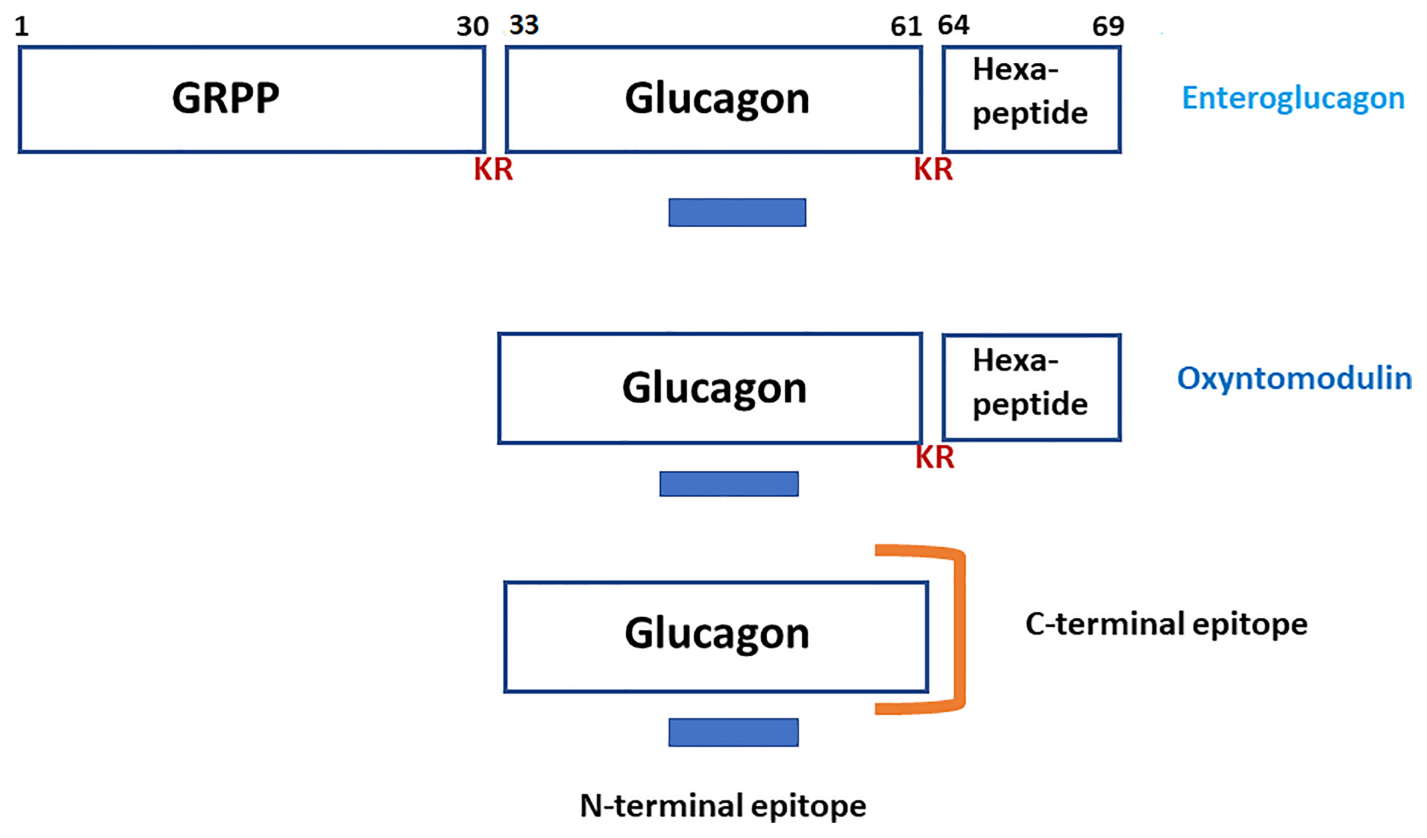
A schematic representation of the structures of enteroglucagon (proglucagon-(1-69), glicentin-related polypeptide [GRPP; proglucagon (1-30)], glucagon [proglucagon (33-61)] and oxyntomodulin [proglucagon (33-69)]. KR represents the dibasic residue processing sites. The blue bar indicates the epitope in the N-terminal to central region of glucagon that is recognized by both “pancreatic glucagon-specific” and “gut GLI cross-reactive” antisera. The orange symbol illustrates the epitope at the C-terminus of glucagon that is recognized by “pancreatic glucagon-specific” antisera only.

Elucidation of the nucleotide sequence of a cDNA encoding human proglucagon (1) established unambiguously that the molecule contained, in addition to glucagon, two additional regions whose primary structures suggested that they represented biologically active peptides. In addition to domains containing glucagon-like peptide-1 [GLP-1; proglucagon-(78-107) amide] and glucagon-like peptide-2 [GLP-2; proglucagon-(126-158)], a domain at the N-terminus of the precursor contains enteroglucagon [proglucagon-(1-69) also known as glicentin and gut glucagon-like immunoreactivity (GLI)]. Within enteroglucagon, the sequences of glicentin-related pancreatic peptide [GRPP; proglucagon-(1-30)], glucagon [proglucagon-(33-61)] and oxyntomodulin [proglucagon-(33-69)] are embedded (**Figure 1**). The story of the discovery of GLP-1 and GLP-2 and the role of the truncated form of GLP-1 [proglucagon (78-107) amide] as a physiologically important incretin and GLP-2 as a trophic factor of intestinal mucosa has been told in several recent comprehensive reviews (2–5) and so does not need re-telling in this article. For the sake of clarity, an indication of the structural relationships between these proglucagon-derived peptides is provided in **Table 1**.

**Table 1:** Structural relationships between proglucagon-derived peptides.

Enteroglucagon	Proglucagon (1-69)
GRPP	Proglucagon (1-30)
Glucagon	Proglucagon (33-61)
Oxynomodulin	Proglucagon (33-69)
GLP-1	Proglucagon (72-107) amide
Truncated GLP-1	Proglucagon (78-107) amide
GLP-2	Proglucagon (126-158)

As a former post-doctoral fellow in the laboratories of Prof. Keith D. Buchanan, Queen’s University of Belfast, N. Ireland (1974-7) and Prof. Roger H. Unger, Southwestern Medical School, Dallas, U.S.A. (1977-9), I will present in this article a somewhat personal interpretation of the early literature relating to enteroglucagon prior to the discovery of GLP-1 and GLP-2 in 1982. This is an attempt to explain how a measure of order was brought to a thoroughly chaotic situation regarding the measurement of GLI concentrations in plasma and tissue extracts as well as how ambiguities regarding structural characterization of the immunoreactive components were resolved. Nevertheless, enteroglucagon and its N-terminal domain, GRPP remain enigmatic to this day in terms of their physiological roles (if any).

## Identification of Enteroglucagon by Radioimmunoassay

The discovery by Kimball and Murlin in 1923 of an hyperglycemic factor in the pancreas, which they termed glucagon, is contemporaneous with the discovery of the hypoglycemic factor, insulin (6) but it was not until 1957 that the primary structure of the hormone was determined by Bromer and co-workers in the Lilly Research Laboratories (7). Subsequent work by the group of Sundby at Novo Nordisk demonstrated that, with the exception of the hystricomorphs (8), the structure of the peptide has been very strongly conserved among mammals including the human (9). The presence of components with glucagon-like hyperglycemic properties in extracts of intestinal mucosa was first reported by Sutherland and De Duve in 1948 (10) but it was the development of the first radioimmunoassay (RIA) for glucagon by the group of Unger in 1961 (11), soon after the first reported RIA for insulin (12), that permitted an investigation of the structure, distribution and properties of enteroglucagon. However, it became apparent that radioimmunoassays for glucagon were associated with several issues that were not observed in applications of the insulin radioimmunoassay.

The demonstration that gastrointestinal tissues contained material that was recognized by antisera raised against glucagon was made by Unger and co-workers in 1966 but they initially concluded that “The results clearly indicate that glucagon or a factor with the same immunochemical characteristics is present in acid-alcohol extracts of gastrointestinal tissues of rat, dog and man.” (13). The observation that glucagon-like material in the alimentary tract was not identical to pancreatic glucagon was made by Samols and co-workers on the basis of non-parallelism of inhibition slopes under RIA conditions (14). This conclusion was confirmed by the demonstration that intraduodenal administration of glucose to dogs resulted in a marked increase in GLI concentration in the mesenteric vein but a decrease in the pancreatic venous effluent and that the material released from the gut was chromatographically different from that released from the pancreas (15).

It soon became apparent that antisera raised against glucagon could be divided into two classes in terms of their ability to recognize the glucagon-like components present in the gastrointestinal tract. Antisera such as the widely distributed Unger antiserum 30K (16) were described as “pancreatic glucagon specific” because they measured only very low concentration of GLI in extracts of intestinal tissues whereas antisera, such as Heding antiserum K4023 (17) were described as “gut GLI cross-reactive” because they detected high concentration of GLI in both pancreatic and intestinal extracts. The molecular basis for this difference in properties was elucidated by structure-immunoreactivity studies using a range of glucagon fragments (18, 19). As shown in **Figure 1**, the glucagon molecule is associated with two antigenic sites. The “GLI cross-reactive” antisera are directed against an epitope in the N-terminal to central region of the peptide (residues 6-15) whereas the “glucagon specific” antisera are directed against an epitope in the C-terminal region of the peptide (residues 24-29) that requires the presence of a free C-terminal carboxyl group for binding to antibody. It was concluded, therefore, that enteroglucagon, like oxyntomodulin, must comprise glucagon extended from its C-terminus by additional amino acid residues. Conversely, circulating components larger than glucagon that were detected with C-terminally directed antisera must represent proglucagon-derived peptides that lack this C-terminal extension (20).

Prior to the cloning era, our understanding of the nature of proglucagon was complicated by reports that human (21) and dog (22) plasma contained varying concentrations of a high molecular mass component described as “big plasma glucagon”. This component was eluted from gel permeation columns in the globulin fraction of plasma (M_r_ >100,000 Da) and was detected by both C-terminally and N-terminally directed antisera. Related contemporary studies also described high molecular mass forms of a number of other peptide hormones (ACTH, calcitonin, gastrin, growth hormone, and LHRH) in human plasma by using the techniques of gel permeation chromatography and radioimmunoassay [reviewed in (23)]. Although the technique of RIA has undoubtedly revolutionized the field of endocrinology, inappropriate interpretation of RIA data has led to the appearance of many articles in the literature for which the kindest description is that they are irreproducible. It is frequently forgotten that RIA does not measure directly the concentration of a component in a biological fluid or tissue extract but the concentration of substances that inhibit the binding of the radiolabeled tracer to antibody. In this light, it became apparent that these “big plasma hormones” represented factors, particularly α- and β-globulins, that non-specifically inhibited binding of radiolabeled tracer to antibody (23, 24). Consequently, it was concluded that removal of these interfering components from plasma prior to assay, for example, by selective ethanol precipitation was strongly advised, if not mandatory, for accurate determination of circulating glucagon concentrations (25). By the same token, [^125^I]-labelled glucagon tracers are rapidly degraded by peptidases in human plasma. The inability of the damaged tracer to bind to antibody may also be erroneously interpreted as the presence of antigen. Similarly, the high molecular mass GLI component reported to be present in extracts of salivary glands was shown to represent an artefact arising from tracer-degrading activity (26). The problem was recognized early and to some extent addressed by the inclusion of the peptidase inhibitor Trasylol (Aprotinin) in the assay buffer (27) but denaturation and precipitation of the degrading enzymes by addition of ethanol is more effective in eliminating this interference (25).

## Structural Characterization of Enteroglucagon

The first suggestion that glucagon, like insulin, may be derived from a biosynthetic precursor larger than itself was provided by Tager and Steiner in 1973 who isolated and characterized chemically a peptide impurity in a sample of bovine/porcine glucagon. This component comprised glucagon extended from its C-terminus by the octapeptide Lys-Arg-Asn-Asn-Lys-Asp-Ile-Ala (28). The authors correctly speculated that the peptide, subsequently shown to be identical to oxyntomodulin isolated from porcine jejuno-ileum (29), represented a fragment of proglucagon.

Prior to the availability of (relatively) affordable and reliable equipment for HPLC in the 1980s, gel permeation chromatography using columns of Sephadex G-50 or Biogel P-10 matrices was the preferred method to purify partially and estimate the molecular mass of peptides. However, the correlation between molecular mass and elution volume is far from exact as interaction of the peptide with the matrix is also dependent to some extent on molecular charge and secondary structure. The major component of GLI in extracts of porcine ileum (30, 31) and colon (32) and in canine brain (33) was eluted from gel permeation chromatography columns with the same elution volume as human cytochrome c (M_r_ 12,225 Da) leading to the incorrect assumption that the molecular mass of enteroglucagon was approximately 12,000 Da. A second component in the extracts (subsequently identified as oxyntomodulin) was eluted in a volume slightly less than that of glucagon indicating a molecular mass of approximately 4,500 Da. The error in estimating the molecular mass of enteroglucagon was compounded by the report based upon amino acid composition analysis that porcine enteroglucagon contained 100 amino acid residues, hence the term glicentin (GLI-cent-in) was adopted (34). The article did, however, lead to the recognition that enteroglucagon contained the full sequence of glucagon and both peptides were derived from the same biosynthetic precursor (35, 36).

Unambiguous determination of the primary structures of porcine enteroglucagon (glicentin) (37) and GRPP (38) was finally achieved by Thim and Moody. As shown in **Figure 1**, glicentin comprises 69, not 100, amino acids (M_r_ 8128 Da) with GRPP constituting the 30 amino-acid-residue N-terminal domain and oxyntomodulin the 37 amino-acid-residue C-terminal domain. For those readers, such as myself, who adopt a pedantic attitude to nomenclature, retention of the term glicentin, especially when referring to enteroglucagon from species other than the pig, is a source of minor irritation.

## The Search for a Biological Role for Enteroglucagon

In contrast to the proglucagon-derived peptides oxyntomodulin, GLP-1 and GLP-2, attempts to establish a well-defined physiological function for enteroglucagon have been beset with problems that still have not been resolved. Initial attempts to determine such a function were hampered by the lack of availability of pure peptide for use in bioassays.

Not long after the discovery of gut GLI, it was shown that an extract of dog jejunum, partially purified by gel permeation chromatography, lacked hyperglycemic activity when injected endoportally into dogs, was devoid of glycogenolytic activity in the isolated perfused rat liver and did not increase hepatic cAMP concentrations (15). The observed glucagon-like ability of the crude extract to stimulate insulin release from the perfused pancreas was presumably a consequence of the presence of GLP-1 and glucose-dependent insulinotropic polypeptide (GIP). Material from porcine ileum (30) and colon (32) that was purified to a higher degree by immunoaffinity chromatography lacked lipolytic activity when incubated with chicken adipocytes, was devoid of insulin-releasing activity in the isolated perfused rat pancreas and did not activate adenylate cyclase in rat hepatocytes or inhibit the binding of [^125^I]glucagon to liver plasma membranes. In addition, the material did not display a cholecystokinin-like ability to stimulate exocrine pancreatic section in the cat or inhibit the stimulatory action of secretin (39).

Of some historical interest was the report that a patient with a renal tumor releasing large amounts of enteroglucagon into the circulation displayed, in addition to colonic and jejunal stasis and malabsorption, marked small intestinal villous hypertrophy and these abnormalities disappeared following resection of the tumor (40, 41). These observations led to speculation that enteroglucagon may be functioning as a growth factor for the intestinal mucosa but it now seems likely that GLP-2 co-released by the tumor was at least partially responsible for the observed trophic effects.

**Figure 2:**
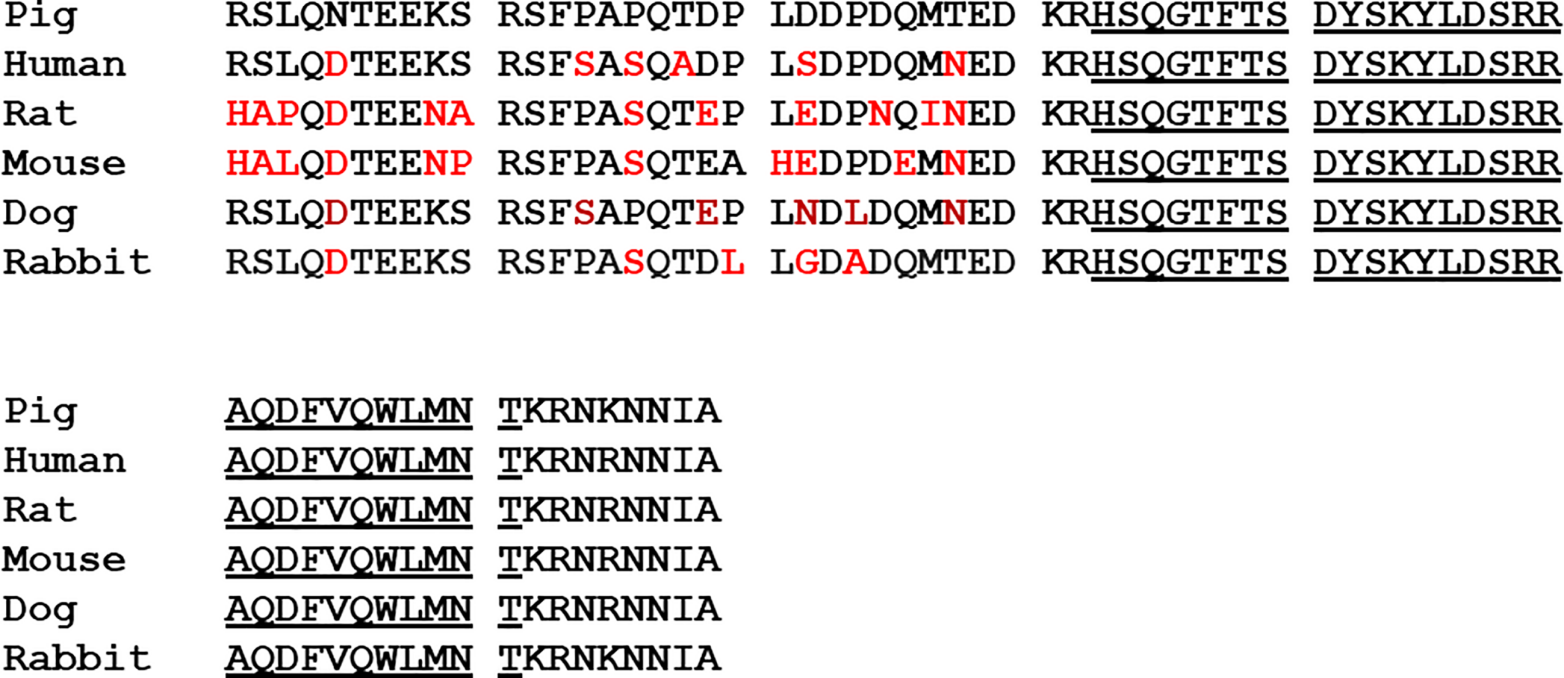
A comparison of the primary structures of pig glicentin with enteroglucagon from human, rat, mouse, dog and rabbit, deduced from the nucleotide sequences of cDNAs. Differences in amino acid sequence from the porcine peptide are shown in red. The glucagon sequence is underlined.

Interest in the physiological function of enteroglucagon was stimulated by the availability of highly purified porcine glicentin provided by Novo Nordisk. However, unambiguous interpretation of data obtained with this material is not always possible. Except in a few cases, experimental studies have been carried out, not in pigs, but in rodents, rabbits and dogs but, in contrast to glucagon and oxyntomodulin, the primary structure of the N-terminal domain of enteroglucagon (GRPP) has been poorly conserved among mammals (**Figure 2**). Consequently, effects observed with glicentin in such bioassays could be regarded as pharmacological rather than physiological and conversely lack of effects may be a consequence of differences in amino acid sequence. Additionally, proteolytic conversion of glicentin to glucagon, glucagon fragments and possibly oxyntomodulin by incubation with rat hepatocytes (42) and enterocytes (43) has been demonstrated so that any glucagon-like biological effects observed may be a consequence of the *in situ* generation of these peptides. For example, glicentin had 1% of the potency of glucagon on glucose production when incubated with hepatocytes but the peptide was concomitantly degraded to smaller components some of which were chromatographically very similar to glucagon and its fragments suggesting that glicentin may exert its glucagon-like effects on hepatocytes only after degradation to smaller glucagon-related peptides (42). Similarly, oxyntomodulin and its (19–37) and (30–37) fragments inhibit histamine-stimulated gastric acid secretion in the rat (44) so that the observation that glicentin infusions inhibit pentagastrin-stimulated gastric acid secretion in the rat is subject to the interpretation that the effect may arise, at least in part, from its conversion into these products (45).

More recent studies using synthetic GRPP and glicentin or with naturally occurring peptides purified to near homogeneity have been comprehensively reviewed by Raffort et al. (46). However, the problem of interpretation of data generated by peptides in heterologous animal models remains. For example, infusion of porcine GRPP and glicentin 1-16 in dogs resulted in an increase in plasma insulin and a decrease in plasma glucagon (47) but rat GRPP elicited potent inhibition of glucose-stimulated insulin secretion from the rat pancreas (48). Inconsistent results have also been reported regarding trophic effects of glicentin on intestinal mucosa and gastrointestinal motility that again may be explained to some extent by species differences (46). The possibility that enteroglucagon may have a function as a barrier-sustaining agent was suggested by the report that recombinant human enteroglucagon inhibited internalization of the bacteria *Salmonella enteritidis*, *Escherichia coli*, and *Enterococcus faecalis* by INT-407 human-derived embryonic intestinal cells (49). This observation may have clinical significance in terms of maintenance of the integrity of tight junctions within in the intestine thereby inhibiting extraintestinal invasion by pathogenic bacteria.

## Conclusion

It must be concluded that, at this time, the physiological role of enteroglucagon remains unclear. A specific receptor for enteroglucagon has not been identified and the fact that evolution has not co-opted the large amounts that are released into the circulation peptide for an hormonal function seems surprising. The pathways of differential processing of proglucagon by prohormone convertases into predominantly GRPP, glucagon, and a large inactive C-terminal fragment in the pancreas and to enteroglucagon/oxyntomodulin, GLP-1 and GLP-2 in the gut are well established (5). It makes little metabolic sense that the mammalian intestine should release equimolar amounts of the hyperglycemic factor, glucagon and the hypoglycemic factor GLP-1 in response to ingested nutrients and so it may be that inactive enteroglucagon simply represent nature’s strategy for obviating the hyperglycemic action of proglucagon-derived peptides in the gut.

## Author Contributions

The author confirms being the sole contributor of this work and has approved it for publication.

## Conflict of Interest

The author declares that the research was conducted in the absence of any commercial or financial relationships that could be construed as a potential conflict of interest.
